# Treatment of Prolapsus Uteri

**Published:** 1871-03

**Authors:** C. A. Spencer

**Affiliations:** Dallas, Pa.


### Treatment of Prolapsus Uteri.

BY C. A. SPENCER, M. D., OF DALLAS, PA.

Re-published by request from "Philadelphia Medical and Surgical Reporter."

Among the many wonderful adaptations of means to an important end, with which the study of anatomy makes us acquainted, not the least remarkable is the contrivance by which the uterus is suspended in the pelvic cavity, so movable as to escape any rude scratch from without or any inconvenience from the varying conditions of the surrounding viscera, and yet so tethered to its place as to insure its enlargement going on, if pregnancy occurs, in such a direction as shall avoid needless discomfort to the person, or pressure upon and disorder of the functions of other organs. But the very mobility, without which pregnacy would be a season of uninterrupted suffering, and even sexual intercourse almost impossible, exposes the womb to the risk of changes in its position, such as may themselves become the source of inconvenience, and call more frequently than almost any other uterine ailments for medical interference. It is obvious enough that an organ suspended within a spacious cavity by means of supports which are themselves yielding, must be very likely to be displaced by comparatively slight causes. In the case of the uterus, too, the risk of its displacement is further increased by the circumstance, that its weight and size are subject to variations, and that the very causes which tend to render it heavier and larger than natural, have often the further effect of diminishing the power of the supports by which it is retained in its natural positions. The tendency to misplacement is further encouraged by the presure from above of the superincumbent viscera, and by all these muscular exertions a person cannot avoid making in walking, in lifting weights, or even in efforts of defecation.

These causes tend to produce displacements upon its own axis, which are the common and frequent result of the accompanying constipations; and are the most frequent causes of displacement symptoms. These patients usually complain of pain in the back, of a beating or throbbing character, headache, sharp pains through the chest, pain in the left iliac region, with a dragging or pulling sensation, feeling of fullness in perineum, frequent but false desire to evacuate the bowels. Irregularity of the menstrual functions, loucorrhœa, irritable bladder, constipation and diarrhœa. On making a vaginal examination, the womb is found lower down than natural, or the cervix pressing against the rectum. The os and cervix are usually enlarged. The os has a velvety feeling with more or less tenderness.

*Treatment.*—The treatment in all cases must be governed bythe condition of the patient. If the case is of long standing, and the system much debilitated and a great relaxation of the much debilitated and great relaxation of the muscles, I have found the following prescription to afford marked relief:

| | | |
|---|---|---|
| ℞. | Fld. ext. nux vomic., - - - - - | f.ʒi. |
| | Fld. ext. bleubahosh, - - - - | fʒss. |
| | Fld. ext. ergot., - - - - - | f.ʒiij. |
| | Comp. ticture cinchona, - - - | f.℥j. |
| | Simple syrup, - - - - - - - | f.ʒiij. M. |

Dose.—One teaspoonful four times per day.

But in all cases where there is prolapsus or other displacement of the uterus, it should be returned to its natural position, and some form of an instrument introduced to retain it there, and the instrument that I prefer is Dr. S. S. Stauffer's Philadelphia Gutta Percha Silver Stem Pessary, advertised in this journal.

I have used them in a number of cases, and in every one they have acted admirably, relieving all of those distressing pains, commonly termed bearing down; and most of the other symptoms, under proper treatment, soon disappear. This instrument is very ingeniously constructed with a cup, which embraces the cervix uteri; this cup rests upon a silver stem, the former inclosed so as to prevent friction against the soft parts. The base rests upon a perineal bandage, which is buckled to an elastic abdominal belt. It can not fail to keep the womb in its most natural position, and the moderate price puts them in reach of every woman wishing them. If there is leucorrœa, the following will relieve it promptly:

℞. Hamamelin, - - - - - - ʒiss.

White sugar, - - - - - - ʒiij.

Ent. ergot, - - - - - - - f.℥ss.

Simple syrup, - - - - - - f.℥iij. M.

Triturate the hamamelin and sugar until they are well mixed, then add the syrup, lastly the ergot, and give one teaspoonful 4 or 5 times per day.

℞. Muriate ammonia,

Sulp. zinc. - - - - - aa. ʒj.

Water, - - - - - - - - Oj. M.

Sig.—Use one syringe full night and morning.

If the uterus is enlarged, bichloride of mercury and bromide of potassium are the remedies. If there is irregularity of the menses, senecin gossypein and iron should be used. And if there should be a lack of nutrition, cod liver oil and port wine are called for.

Case I.—Miss A. K., aged 18, taken sick Feb. 24th, 1870. I was called Feb. 25th; found her with great pain in back and region of the uterus, with pains and soreness in the left iliac region. When she attempted to stand on her feet (using her own words) she had such a bearing down, and such a dreadful feeling in her back, that she was compelled to lie down again. On examining per vagina, I found the cervix uteri restingon the perineum, the uterus enlarged, very hot and tender. She stated that she had not menstruated in about eight weeks. Treat senecin, gr. 20; gossypiir gr. 20; feri. phos., gr. 10; mix chart No. 7; give one every four hours. Morphia sulph., gr. 2; fld. ext. belladonna, gtt. 30; aqua camphor, ℥ii; sig. teaspoonful every half hour, until the pains relieved. Feb. 26th, 2 p. m., found her comfortable with free menstrual discharge. Potass. bromide, ʒi; hydrarg. brichlori, gr. 1; cypripedir, ʒi; syr. simple, ℥iv; sig. teaspoonful four times per day. Ten days later, on examination, found the uterus natural size, not tender, and easily returned to its natural position. I introduced one of Stauffer's shell pessaary's, and gave her a tonic. March 28th, removed the pessary and told her to walk around the room, which she did. On examining after, I found the uterus nearly in its natural position. I again introduced the pessary, and did not see her again until June 12th, when she told me she had not worn the instrument for the past six weeks, and that she had not felt any of the old complaint, although she had worked very hard.

CASE II.—Miss Lidy A., age 22 years, school teacher, single, had been unwell for two years, her physicians telling her she had rheumatism, as she had pains in her limbs and through her chest and back. She stated that she had not been able to teach for the past year. She applied to me for treatment March 28th, 1870. After hearing her history, I told her I thought she had falling of the womb. She said she thought that could not be; she did not think she was sick enough for that; but she consented to be examined, which revealed procidentia uteri. I advised her to get one of Dr. Stauffer's instruments, which I accordingly obtained for her in a few days. I used in this case a shell pessary, as above. The medical treatment consisted of uterine tonics with nervines, and with orders to push the instrument up every night after retiring to bed. The first of May, her health having improved, she commenced her school again, which she has continued up to the present time, Dec. 27th, except a vacation of October and November. I saw her during the vacation, when she reported herself as feeling well in every respect, and stated that she had not worn the instrument in about two months. On making examination I found the uterus in its natural position, and to all appearances healthy.

CASE III.—Mrs. C. G., age 28, married, has two children, the youngest 4 years old. She states that her last confinement was tedious and protracted, and that ever since, whenever she works hard her womb comes down and out about two inches, and to returne it she is compelled to lie down on the floor with her feet upon the lounge or chair and work it back, and then after lying in that position for a short time it will stay up until lifting or some other over work brings it down again. I advised her to have an instrument to keep it in position. I accordingly procured for her one of Dr. Staufers Gutta Percha Silver Stem Pessary, and returned the womb to its natural position, then introduced the instrument to keep it there, and gave her uterine and muscular with general tonics, such as nux vomica, hydrostin tr. cinchona comp., iron, &c., for about two months when the medicine was discontinued. She reporting herself as feeling well, and had gained about fifteen pounds of flesh. Dec. 23d, saw her; she reports herself well; says she has not worn the instrument since October first, and that she has not felt any of the old disease in about four months.

CASE IV.—Mrs. J. K., married, has two children. I was called to see her August 24th, 1870. She stated that she had not been able to do her own house-work in about two years; that sometimes she felt quite well, and then she would try to work, but that always made her worse. At the time I first saw her she had menorrhagia with prolapsus uteri. She was pale and anæmie. The medical treatment after of tonics, bark, iron, &c. Sept. 10th, introduced one of Dr. Stauffer's silver stem pessaries, and in about four weeks she discharged her servant girl, and has done her own work ever since, and now looks ten years younger than she did when I commenced treating her, and says she feels better than she ever remembers of feeling before. I do not mean to say that Dr. Stauffer's instruments will cure all cases of prolapsus uteri, but that the majority of uncomplicated cases in females before menstruation ceases, with those instruments and appropriate medical treatment, I am fully persuaded by my own as well as other physician's experience. In young females I use the shell or globe pessary; and in married, with lax muscular habit, I use the silver stem pessary.

